# ARIMA modelling and forecasting of irregularly patterned COVID-19 outbreaks using Japanese and South Korean data

**DOI:** 10.1016/j.dib.2020.105779

**Published:** 2020-05-26

**Authors:** Xingde Duan, Xiaolei Zhang

**Affiliations:** a School of Mathematics and Statistics, Guizhou University of Finance and Economics, Guiyang, P. R. China; b Pan-Asia Business School Yunnan Normal University, P.R. China

**Keywords:** Daily new cases, Statistical analysis, stationarity, Dynamic prediction

## Abstract

The World Health Organization (WHO) upgraded the status of the coronavirus disease 2019 (COVID-19) outbreak from epidemic to global pandemic on March 11, 2020. Various mathematical and statistical models have been proposed to predict the spread of COVID-2019 [1]. We collated data on daily new confirmed cases of the COVID-19 outbreaks in Japan and South Korea from January 20, 2020 to April 26, 2020. Auto Regressive Integrated Moving Average (ARIMA) model were introduced to analyze two data sets and predict the daily new confirmed cases for the 7-day period from April 27, 2020 to May 3, 2020. Also, the forecasting results and both data sets are provided.

Specifications Table

**Table:** 

**Subject**	Infectious Diseases
**Specific subject area**	ARIMA model applied to predict COVID-19 outbreaks
**Type of data**	Table Image
**How data were acquired**	The data on daily new confirmed cases of COVID-19 were taken from Wind Database. The data ware built as a time-series database by excel 2017 and ARIMA model was established for analysis using R software.
**Data format**	Raw
**Parameters for data collection**	Under the framework of Box-Jenkins method, model identification, estimation, diagnostic checking, and forecasting for ARIMA model was applied to the daily new confirmed cases data in Japan and South Korea.
**Description of data collection**	The daily new confirmed cases data of the COVID-19 outbreaks in Japan and South Korea from January 20, 2020 to April 26, 2020 are available from the Wind Database(https://www.wind.com.cn/newsite/edb.html). Also, there are no missing values and the Excel file of the daily data are presented in Supplementary data.
**Data source location**	Japan and Korea
**Data accessibility**	With the article The raw data is in Appendix A.

**Value of the Data**

• These data are easy to collect, and countries are beginning to collect and collate the data and release it publicly for study and analysis.
• These data can be updated through news and websites to facilitate tracking and analysis during the development of the epidemic. In particular, data on daily new cases are useful because they can be used to predict covid-19 outbreaks. The data from these two typical Asian countries have practical implications for the analysis and intervention of covid-19.
• The analysis of new data with ARIMA model can timely analyse and predict the changes of COVID-19, and provide dynamic information to relevant departments.
• At the same time, other research institutions and management departments can also use these data to timely track and study the development and changes of the epidemic.

## 1 Data Description

**Figure 1:**
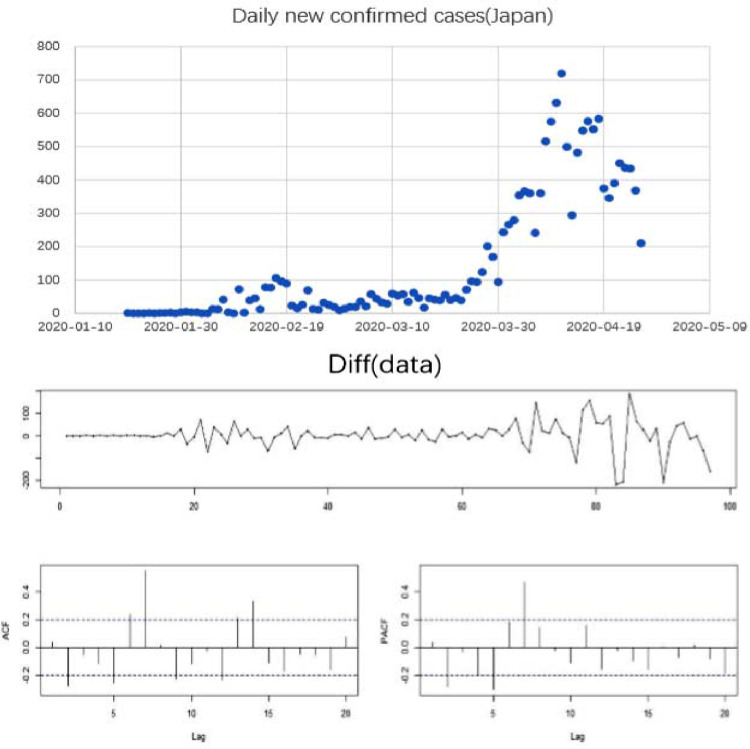
Daily new confirmed cases in Japan,first- difference of the original data ,ACF and PACF PLOT.

**Figure 2:**
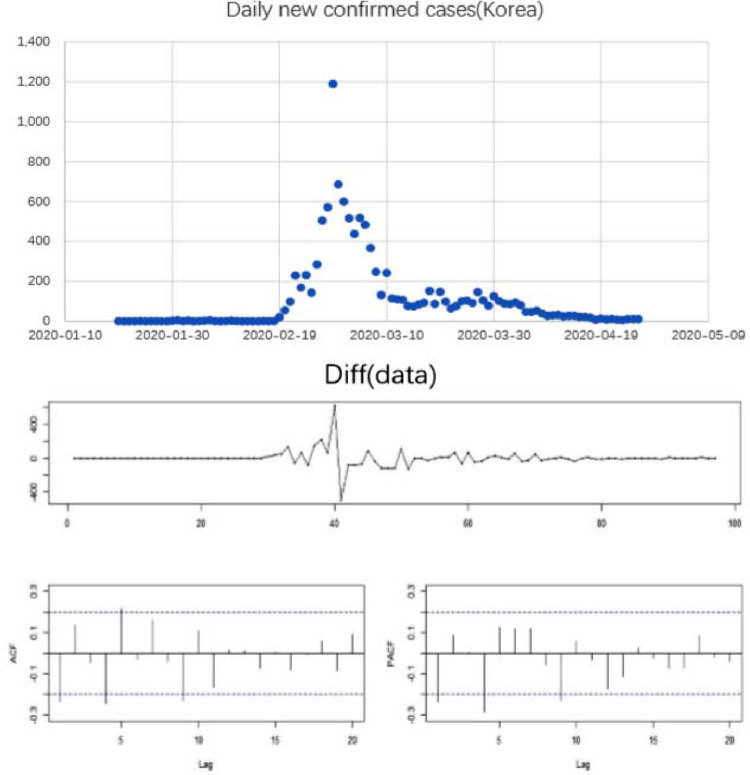
Daily new confirmed cases in South Korea,first- difference of the original data ,ACF and PACF PLOT.

The daily new confirmed cases data of the COVID-19 outbreaks in Japan and South Korea from January 20, 2020 to April 26, 2020 are available from the Wind Database[2]. Also, there are no missing values and the Excel file of the daily data are presented in Supplementary data. The data were analysed using the statistical software R. To visualize the data time series plots of the daily new confirmed cases data in Japan and South Korea for the 98-day period from January 20, 2020 to April 26, 2020, are displayed in Figure 1, Figure 2; respectively. It can be seen from Figure 1 and 2 that both original time series look much more nonstationary and present irregular pattern; therefore, the differencing transformation was incorporated as a useful approach to stabilize the original time series. In addition, the first- difference time series look stationary when compared with the original time series shown in Figure 1 and 2.

## 2 Experimental Design, Materials, and Methods

Auto Regressive Integrated Moving Average model, referred as ARIMA model, is employed to analyse the daily new confirmed cases data in Japan and South Korea; respectively. Under the framework of Box-Jenkins method, model identification, estimation, diagnostic checking, and forecasting for ARIMA model was applied to the two original time series [3,4]. The differencing transformation was used to achieve stationarity on certain nonstationary time series. The Augmented Dickey-Fuller (ADF) unit-root test was also introduced to identify whether the time series is stationary [3]. In addition, the R package “tseries” and “forecast” were implemented to produce the numerical output for ARIMA [5].

**Figure 3:**
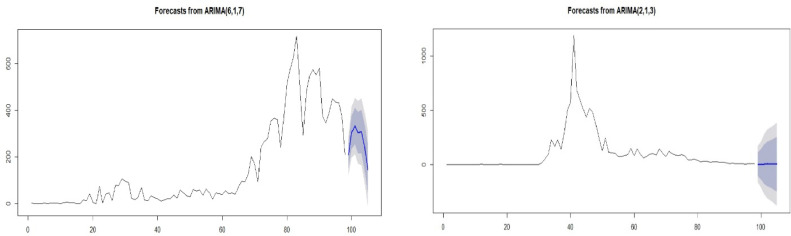
7-day period prediction of the daily new confirmed cases for Japan and Korea plot.

The first difference of two original series and their ADF unit-root test appear to support stationary ARMA model; therefore, we consider a class of stationary ARMA model as appropriate. Combinating parsimonious parameter models, auto-selection of model order based on R package “tseries” and correlogram of the sample autocorrelation function (ACF) and partial autocorrelation function (PACF) shown in Figure 1 and 2, we chose the orders for ARIMA model as ARIMA (6,1,7) in Japan and ARIMA (2,1,3) in South Korea; respectively. Furthermore, we adopted the following moment method and unconditional least squares to estimate the parameters for the stationary ARMA model. To save space, these estimated results were not reported. To check on the independence of the noise terms from the above ARMA model, we implemented the following diagnostic checking tools: a sequence plot of the residuals, the sample ACF of the residuals, and p-values for the Ljung-Box test statistic for a whole range of the residuals; which indicate the residuals from these ARIMA follow the white noise process. Therefore, the estimated ARIMA model can capture the dependent structure of the daily new confirmed cases time series very well. Finally, based on the above ARIMA model, the predicted value and the upper and lower limits of the predicted value under the 95% confidence level of the daily new confirmed cases for the 7-day period from April 27, 2020 to May 3, 2020 were reported in Table 1 and displayed in Figure 3.

**Table 1:** Predicted value under the 95% confidence level of the daily new confirmed cases for the 7-day period

Japan	date	lowwer	mean	upper	Korea	date	lowwer	mean	upper
	2020-04-27	122.68342	207.5012	292.319		2020-04-27	-161.9685	6.36643	174.7014
	2020-04-28	194.68068	303.4768	412.2729		2020-04-28	-210.9135	2.035784	214.9851
	2020-04-29	211.76786	333.6616	455.5554		2020-04-29	-272.6349	4.635792	281.9065
	2020-04-30	170.06375	304.8661	439.6684		2020-04-30	-308.2444	7.649637	323.5437
	2020-05-01	164.28963	308.5206	452.7516		2020-05-01	-330.465	7.153191	344.7714
	2020-05-02	93.39579	244.7979	396.1999		2020-05-02	-354.1099	5.432586	364.975
	2020-05-03	-22.66019	143.4524	309.565		2020-05-03	-381.2896	5.198718	391.687

## Appendix A. Supplementary data

Appendix A.xlsx

## Declaration of Competing Interest

The authors declare that they have no known competing financial interests or personal relationships which have, or could be perceived to have, influenced the work reported in this article.

## Appendix Supplementary materials

Image, application 1

Image, application 2

Image, application 3
